# Epidemiological characterization of tibial plateau fractures

**DOI:** 10.1186/s13018-022-02988-8

**Published:** 2022-02-19

**Authors:** Juan Reátiga Aguilar, Ximena Rios, Eduardo González Edery, Alfredo De La Rosa, Laura Arzuza Ortega

**Affiliations:** 1 Orthopedics and Traumatology Department, Grupo Campbell, Barranquilla, Colombia; 2 Orthopedics and Traumatology Department, Clínica Valle Salud Sur Cali, Valle del Cauca, Colombia; 3 Orthopedics and Traumatology Department, Clinica Bahia, Santa Marta, Colombia; 4 Magister Epidemiology, Grupo Campbell, Barranquilla, Colombia

**Keywords:** tibial fracture, Tibial plateaus, Trauma mechanisms

## Abstract

**Background:**

Tibial plateau fractures are traumatic injuries with severities ranging from nondisplaced to complicated fractures. This study describes the epidemiological characteristics of patients with tibial plateau fractures treated in five trauma clinics.

**Methods:**

This retrospective, cross-sectional study included 1165 patients with tibial plateau fractures treated between December 2015 and May 2017. Subjects were selected from the medical records of five institutions based on the inclusion and exclusion criteria. Age, sex, laterality, fracture type, trauma mechanism, vehicle type, classification, and associated injuries were assessed via univariate and bivariate analyses.

**Results:**

In total, 23.3% of patients with tibial fractures treated during the study period had tibial plateau fractures. Of those affected, 73% were men and 50% were younger than 40 years. Furthermore, 95.7% of fractures were caused by traffic accidents, 82.6% of which involved motorcycles. Fractures were closed in 93.1% of cases, and 78% of subjects had associated injuries. The most common fractures, according to Schatzker classification, were type VI (23%) and V (19.1%) fractures.

**Conclusions:**

Tibial plateau fractures are frequent injuries in our setting and mostly occur in men in their 30 s and 40 s. These fractures are typically caused by motorcycle traffic accidents.

Level of Evidence.

IV.

## Background

Tibial plateau fractures are an important group of traumatic pathologies; their high frequency in recent years and the severity of complications present challenges for orthopedists [1, 2]. Their reported incidence is approximately 1% of all fractures and 8% of fractures among elderly people [3].

The term “pilon” was first used in 1911 by the radiologist Étienne Destot to describe “explosive injuries with the talus impacting the tibia like a hammer hits a nail” [4]. In 1950, Bonin used the term “plafond” (ceiling) to describe joint fractures resulting from the impact of the talus on the tibia due to an axial load [5].

Tibial plateau fractures manifest with various morphological patterns [6], ranging from nondisplaced closed fractures to complicated fractures with significant soft tissue and neurovascular damage that can compromise lower limb viability and require complex and extensive treatment [7].

Variations in the clinical presentation of fractures depend on the force of the impact, age of the patient, and degree of knee flexion at the time of injury [7]. These are important parameters for the initial clinical assessment of fracture classification and the selection of appropriate therapeutic approach [8].

Functional results can range from full recovery to gait disturbances and complications, which increase the functional disabilities and incapacities of patients and affect their well-being and productive capacity, thereby increasing health-related costs [8].

Complications occur in 13–88% of cases of tibial plateau fractures [1, 9]. The most common complications include the following: superficial and deep infections in 6.4–16.1% of cases [3, 8, 10], ligament injuries in 20–30% of cases, meniscal injuries in 10–47% of cases, posterior tibial nerve and common peroneal nerve involvement in 2–4% of cases [8], and deep vein thrombosis in 1.8–5.6% of cases [1, 10]. Pseudarthrosis of the metaphyseal–diaphyseal junction, joint stiffness with loss of lower limb mobility, and posttraumatic osteoarthritis are among the late-onset complications of these fractures [4]. The incidence of posttraumatic osteoarthritis has been reported to be particularly high, and Jagdev et al. [11], Manidakis et al. [10], and Vega et al. [8] identified osteoarthritis in 73.34%, 26.4%, and 24% of cases, respectively.

The increased frequency of tibial plateau fractures and their multiple complications highlight the high impact of this pathology on public health. Therefore, the specific characteristics of these patients must be determined. Accordingly, this study aimed to describe the epidemiological characteristics of patients diagnosed with tibial plateau fractures.

## Methods

This descriptive, retrospective, cross-sectional study included 1165 patients with tibial plateau fractures treated between December 1, 2015, and May 31, 2017, at five Colombian clinics.

The participants were selected by searching the medical records in the information systems of the respective institutions for the codes CUPS 773705, SOAT 13580, and tibial osteosynthesis, which led to the identification of 4426 patients with tibial fractures treated during this period. The clinical histories of these patients were reviewed, and 1165 cases were selected based on the inclusion and exclusion criteria.

The inclusion criteria were a diagnosis of tibial plateau fracture confirmed by X-ray, tomography, or an orthopedic doctor with complete records of the variables assessed in this study. Cases with immature skeletons, metabolic bone disease, and isolated tibial spine fractures and those without knee tomography in their diagnostic examinations were excluded from this study.

This study was approved by the institutional ethics committee in accordance with the current regulations under Resolution 8430 of 1993 of the Ministry of Health of Colombia considering that this work presented no risk to the participants. This study complied with the principles of the Declaration of Helsinki and the International Council for Harmonisation of Technical Requirements for Pharmaceuticals for Human Use, thereby respecting the dignity and protecting the rights and well-being of the people. As a no-risk study, informed consent was not required.

The collected data were processed using IBM SPSS Statistics for Windows, version 25.0. For this purpose, an Excel database was constructed, describing the absolute and relative frequencies of the study variables as well as the measures of central tendency and dispersion. Depending on the variable, normality tests were performed using Kolmogorov–Smirnov test. Chi-squared and Wilcoxon–Mann–Whitney tests were used for comparisons. Statistical significance was indicated by *p* < 0.05, and the parameters were estimated with a 95.0% confidence level.

The variables considered for the analysis included both sociodemographic factors (age and sex, characteristics of the affected individual, and type of vehicle involved) and clinical factors (trauma mechanism, fracture type, injury laterality, associated injuries, and fracture classification).

Tibial plateau fractures were classified according to Schatzker classification [12] following radiological and morphological evaluation. This classification includes six types of fractures, with each numerical increase representing an increase in injury severity; after determining the injury severity, treatment plans were established [2] (Table 1).

**Table 1:** Schatzker classification system of tibial plateau fractures

Fracture type	Condylar fracture pattern	Description
Type I	Lateral condyle fracture	The lateral femoral condyle is driven into the articular surface of the tibial plateau. Shearing fracture pattern that is split off and displaced outwards and Downward
Type II	Lateral condyle fracture	Lateral wedge split with an articular surface depression of the lateral condyle
Type III	Lateral condyle fracture	Lateral condyle depressed fracture without split
Type IV	Medial condyle fracture	Any fracture patterns that affect only the medial condyle
Type V	Bicondylar fracture	Both tibial plateaus are fractured. The fracture line often has the appearance of an inverted Y. There may be an associated fracture of the intercondylar eminence
Type VI	Dissociation of the tibial metaphysis and diaphysis	Transverse or oblique fracture of the proximal tibia which results in dissociation of metaphysis from the diaphysis with varying degrees of comminution of one or of both tibial condyles and articular surface

*Source*: Schatzker et al. “The Tibial Plateau Fracture. The Toronto Experience 1968–1975”

## Results

In total, 23.3% (1.165) of patients with tibial fractures treated in the five trauma clinics during the study period had tibial plateau fractures.

Table 1 shows that a higher proportion of these fractures occurred in men (73%) than in women (27%); moreover, 50% of the affected individuals were younger than 40 years of age (interquartile range = 20). Furthermore, 95.7% of the fractures were caused by traffic accidents, 82.6% of which were associated with motorcycles. Closed fractures were the most common fracture type, accounting for 93.1% of the fractures, with 53% of the fractures occurring in the left leg and 78% of the subjects presenting with fracture-associated ligament and meniscal injuries.

**Fig. 1:**
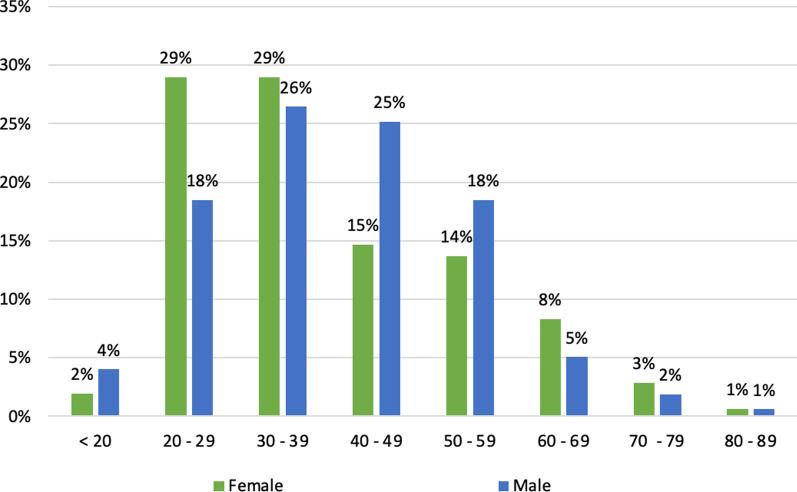
Percentage of tibial plateau fractures by age and gender. Data taken from investigations group database—December 2015 to May 2017

**Fig. 2:**
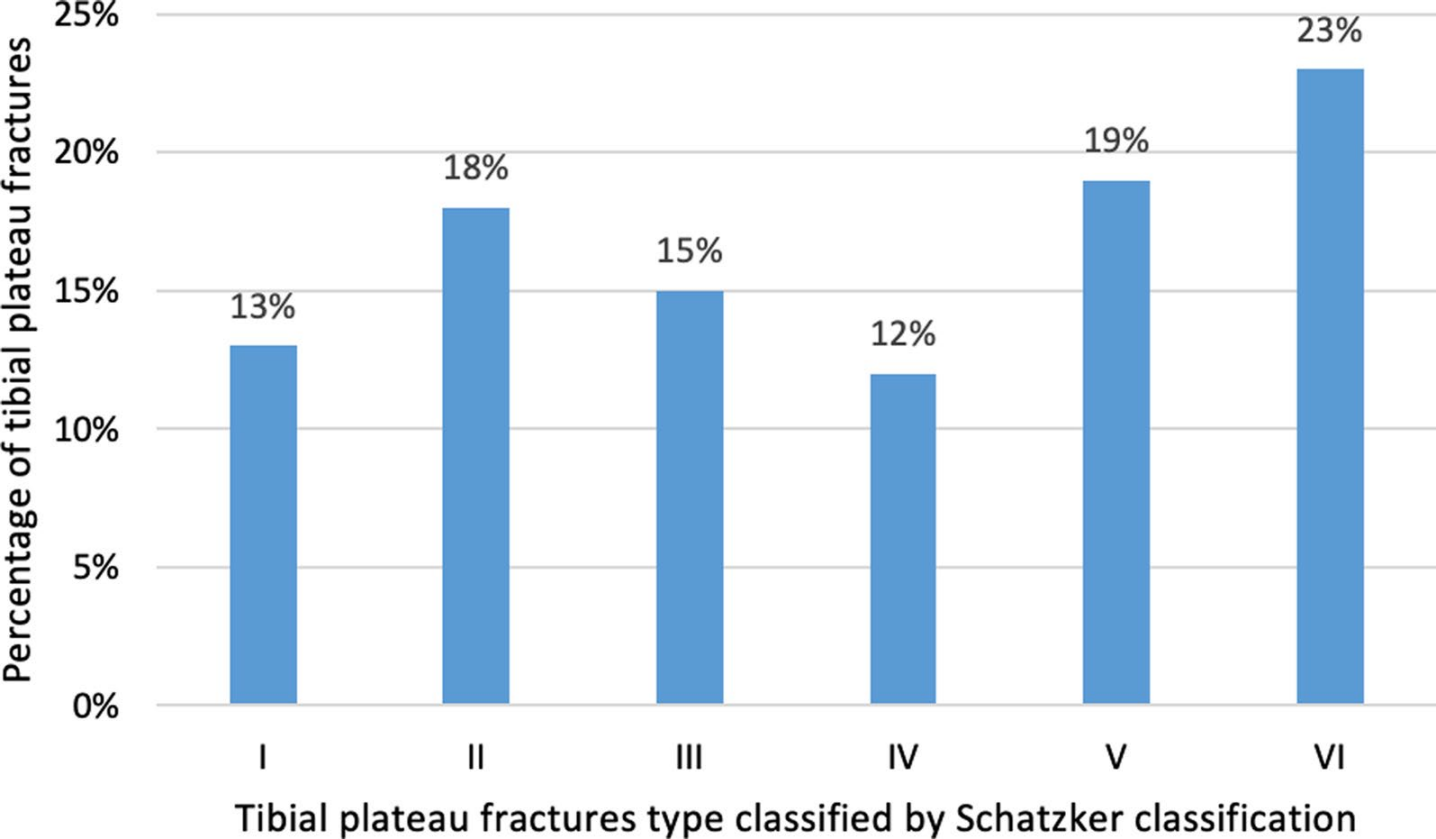
Percent distribution of tibial plateau fractures by Schatzker classification. Data taken from investigations group database—December 2015 to May 2017

Figure 1 shows that in women, these fractures were most common among those aged 20–29 years and 30–39 years (29%). Conversely, in men, they were most common among those aged 30–39 years (26%) and 40–49 years (25%). Traffic accidents resulted in a higher number of tibial plateau fractures in men (73.3%) than in women (26.7%) (*p* = 0.03). Similarly, men had a higher number of associated meniscal and ligament injuries than women (75.3%) (*p* = 0.003). No significant differences in other prevalence rates between sexes were observed (Table 2). The most common tibial plateau fractures were Schatzker type VI (23%), type V (19%), and type II (18.1%) (Fig. 2).

**Table 2:** Demographic characteristics

Variable	Category	*n* = 1165	%
Gender	Male	851	73.0
	Female	314	27.0
Extremity	Left		
Extremity	Right	618	53.0
Fracture type	Closed fracture	547	47.0
Fracture type	Open fracture	1085	93.1
Mechanism of injury	Work accident	80	6.9
Mechanism of injury	Fall from a height	3	0.3
*Vehicle type	Road crash accident	31	2.7
	Sport-related injury	1115	95.7
	Blunt trauma	1	0.1
	Others	1	0.1
	Motor bicycle	14	1.2
	Bicycle	921	82.6
	Car	33	3.0
Associated injuries	Present	161	14.4
Of soft tissues	Absent	914	78.5
		251	21.5

Data taken from investigations group database—December 2015 to May 2017

**Fig. 3:**
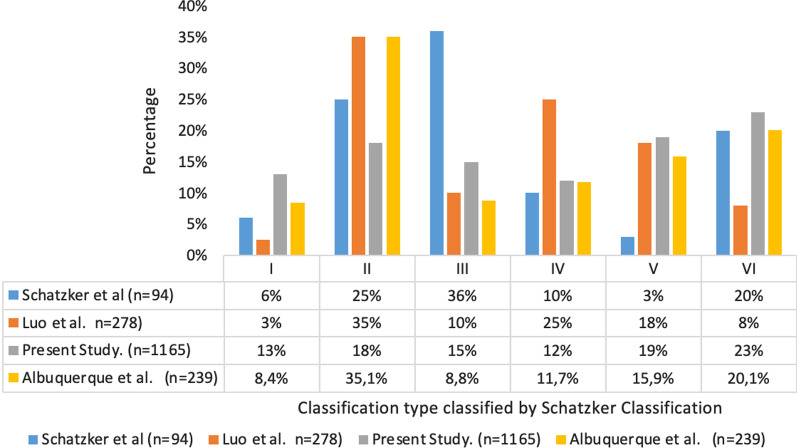
Percentage proportion comparison of Schatzker classification fracture type in the published literature. Data taken from investigations group database—December 2015 to May 2017

Figure 3 presents the percentages of fracture types reported by other authors, including Schatzker in 1975 [12], Zhu et al. in 2012 [13], and Albuquerque et al. in 2013 [14], as well as those observed in this study. Schatzker reported a higher frequency of low-energy type II (25%) and III (36%) fractures, similar to Zhu et al., who also reported a higher prevalence of low-energy type II (35%) and IV (25%) fractures. Albuquerque et al. reported a higher frequency of type II (35.1%) and VI (20.1%) fractures, whereas high-energy type VI (23%) and V (19%) fractures were the most common in the present study (Table 3).

**Table 3:** Prevalence of tibial plateau fractures

		Female	Male	*p* value
Total		314(27%)	851(73%)	
Age (median, RI)	39 RI = 20	35 RI = 20	40 RI = 22	0.01
Extremity	Left	165 (26.7%)	453 (73.3%)	0.84
	Right	149 (27.2%)	398 (72.8%)	
Fracture type	Closed	298 (27.5%)	787 (72.5%)	0.14
	Open	16 (20%)	64 (80%)	
Mechanism of injury	Road crash	298 (26.7%)	817 (73.3%)	0.03
	Work	0	3 (100%)	
	Accident fall from a height	14 (45.2%)	17 (54.8%)	
	Sport injury	1 (100%)	0	
	Others	1 (7.1%)	13 (92.85%)	
	Blunt trauma	0	1 (100%)	
*Vehicle type	Bicycle	7 (24.1%)	22 (75.9%)	0.12
	Motor	235 (25.5%)	686 (74.5%)	
	Bicycle car	56 (33.9%)	109 (66.1%)	
Soft tissue Injuries (Meniscal o Ligamentous Tears)	Present	226 (24.7%)	688 (75.3)	0.001
	Absent	88 (35.1%)	163 (64.9%)	
Classification (Schatzker et al.)	Type I	37 (25%)	111 (75%)	0.6
	Type II	62 (29.4%)	149 (70.6%)	
	Type III	54 (30%)	126 (70%)	
	Type IV	40 (27.6%)	105 (72.4%)	
	Type V	51 (23%)	171 (77%)	
	Type VI	70 (27%)	189 (73%)	

Data taken from investigations group database—December 2015 to May 2017

**Fig. 4:**
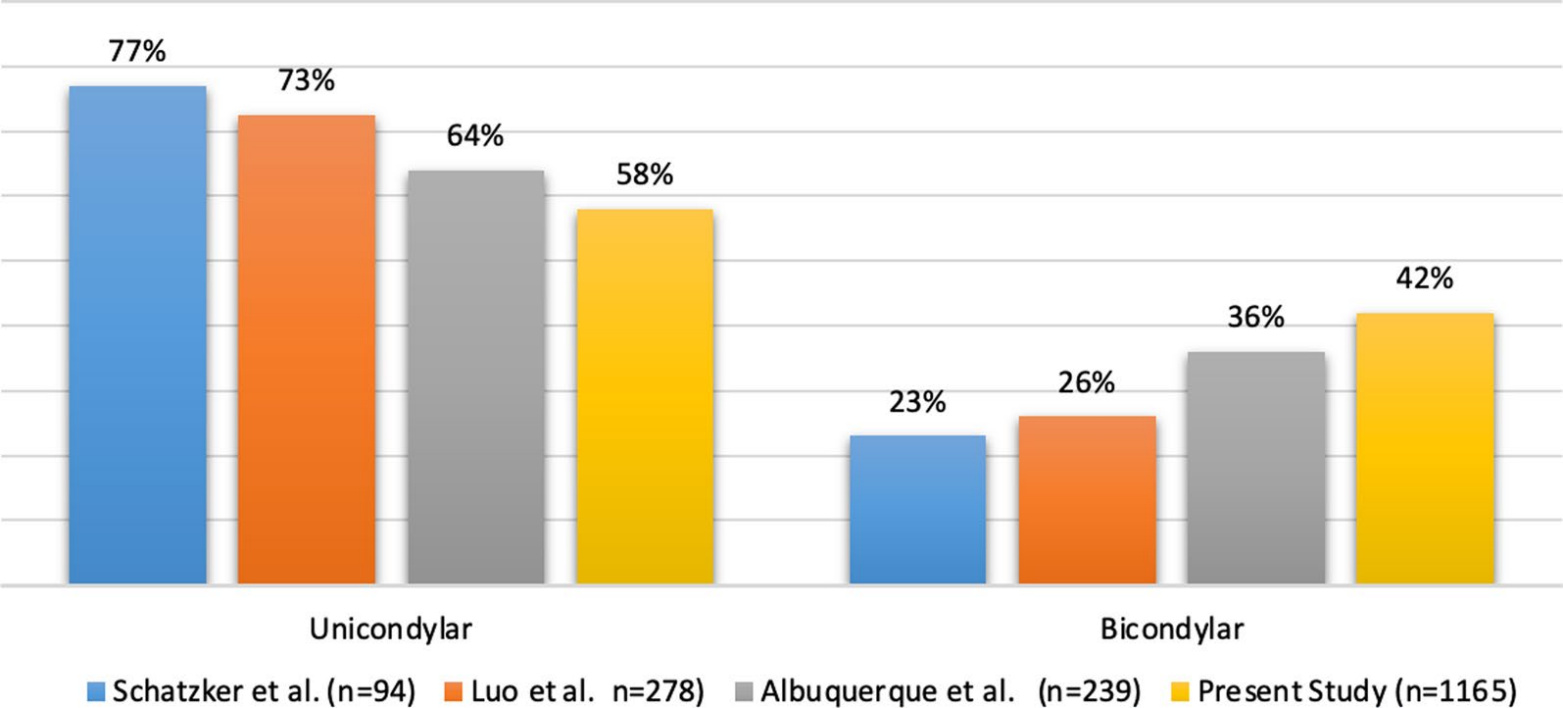
Percentage proportion comparison of tibial plateau fractures classified by condylar involvement reported in the literature. Data taken from investigations group database—December 2015 to May 2017

Figure 4 shows the percentages of tibial plateau fractures based on the degree of severity reported previously and in the present study, highlighting the higher percentages of bicondylar fractures in this study (42%) than those reported by Schatzker et al. (23%) [12], Zhu et al. (26%) [13], and Albuquerque et al. (36%) [14].

## Discussion

The percentage of tibial plateau fractures observed in this study is higher than that reported in the literature [2, 3]. This shows that this type of injury is common in our setting, most likely as a result of the increase in traffic accidents in Colombia in recent years, which is related to the increased use of motorized vehicles, particularly motorcycles, and the risky acts of drivers [3, 8, 13, 14]. A Forensis report revealed that 45,806 people were injured in traffic accidents in Colombia in 2015, of which 54.9% involved motorcycles [15]. In 2018, the National Road Safety Observatory (Observatorio Nacional de Seguridad Vial; ONSV) reported that 6476 people died and 37,213 people were injured in road accidents in Colombia and that the most affected victims were motorcycle users, accounting for 48.3% of the total deaths and 56% of the total number of injured people [14].

In our study, these fractures primarily affected younger people in their 30 s and 40 s, who were economically active and mostly men, corroborating the reports of Vega et al. [8] in Bogotá, Colombia, in 2013 and Albuquerque et al. [14] in Brazil in 2013. These findings could be explained by the higher exposure of young men to traffic accidents according to ONSV, with a ratio of approximately 1.6 men for every woman injured in traffic accidents [16]. These accidents decrease patients’ productive capacity and increase costs resulting from disabilities and rehabilitation treatment.

Another key finding of this study was the higher percentage of high-energy Schatzker type VI and V tibial plateau fractures, with a ratio between the percentages of high- and low-energy fractures being 58/42, which was in contrast to the ratios of 77/23, 73/26, and 64/36 in the studies by Schatzker et al. [12], Zhu et al. [13], and Albuquerque et al. [14], respectively. The results highlight the considerable increase in high-energy fractures in our setting. These fractures are characterized by compromised tibial plateaus and increased soft tissue injury around the knee [14], which are closely related to the high-energy trauma mechanism.

In our study, the percentage of bicondylar injuries was higher (42%) than that reported by Schatzker et al. (23%) [12], Zhu et al. (26%) [13], and Albuquerque et al. (36%) [14], demonstrating the variations in this type of fracture. The increase in more complex injuries has a high impact on public health owing to their serious complications, which require management in specialized trauma care centers for a timely and accurate diagnosis to establish adequate treatment and rehabilitation strategies [3].

## Conclusions

Tibial plateau fractures are common injuries in our setting, primarily occurring in men in their 30 s and 40 s, and are most often caused by motorcycle traffic accidents.

The frequency of high-energy Schatzker V and VI fractures was higher in this study than in other published studies.

Because of the complexity of these injuries, their management in specialized trauma centers should be considered under comprehensive fracture management strategies, guaranteeing a long-term follow-up of patients undergoing surgical treatment for tibial plateau fractures.

## Abbreviation

ONSV: Observatorio Nacional de Seguridad Vial

## References

[1] Cuéllar-Avaroma A, King-Martínez AC, Hernández-Salgado A, Torres-González R (2006). Complicaciones en las fracturas complejas de la meseta tibial y factores asociados. Cir Ciruj.

[2] Álvarez LA, García L, Gutiérrez BM, Montanchez SD (2010). Clasificación de Schatzker en las fracturas de la meseta tibial. AMC.

[3] Carredano X, Valderrama J, Marín F, Valderrama I, Espinoza G (2016). Complicaciones en fracturas de platillos tibiales de alta energía. Rev Chil Ortop Traumatol.

[4] Cutillas IM (2009). Valoración de resultados de las fracturas del pilon tibial. Tesis de grado.

[5] Ortíz P, Allende B, Allende B (1970). Fracturas del pilón tibial ¿Cómo repercuten en la calidad de vida?. Rev Asoc Argent Orton Traumatol.

[6] Allende DP, Scheu G, Carredano G, Colmenares S, Yáñez L, Donoso M (2018). Principios quirúrgicos en fracturas de platillos tibiales con compromiso de columna posterior. Rev Chil Ortop Traumatol.

[7] Robledo-Herrera O, Diego-Ball D, Oliva-Ramírez S (2015). Abordaje posteromedial y colocación de placa en fractura de meseta tibial con fragmento posterior. Acta Ortop Mex.

[8] Vega-Caicedo R, Piñeros-Ramírez DF, Galván-Villamarín F, Medina-Castiblanco C (2013). Descripción epidemiológica y evaluación de los desenlaces de interés de las fracturas de platillos tibiales. Rev Facult Med.

[9] Borade A, Kempegowda H, Richard R, Graham J, Suk M, Horwitz DS (2017). Is “early total care” a safe and effective alternative to “staged protocol” for the treatment of Schatzker IV-VI tibial plateau fractures in patients older than 50 years?. J Orthop Trauma.

[10] Manidakis N, Dosani A, Dimitriou R, Stengel D, Matthews S, Giannoudis P (2010). Fracturas de la meseta tibial: resultado funcional e incidencia de osteoartritis en 125 casos. Ortop Int.

[11] Jagdev S, Pathak S, Kanani H, Salunke A (2018). Functional outcome and incidence of osteoarthritis in operated tibial plateau fractures. Arch Bone Jt Surg.

[12] Schatzker J, Mcbroom R, Bruce D (1979). The tibial plateau fracture. The Toronto experience 1968–1975. Clin Orthop Relat Res.

[13] Zhu Y, Yang G, Luo C, Smith W, Hu C, Gao H (2012). Computed tomography-based three-column classification. J Trauma Acute Care Surg.

[14] Albuquerque R, Hara R, Prado J, Schiavo L, Giordano V, Amaral N, et al. Estudo epidemiológico das fraturas do planalto tibial em hospital de trauma nível I. [electrónicos]. Rio de Janeiro. 2013. Accessed 17 Oct 2017.

[15] Instituto Nacional de Medicina Legal y Ciencias Forenses. Grupo Centro de Referencia Nacional sobre Violencia. Comportamiento de muertes y lesiones por accidentes de transporte, Colombia 2015. Bogotá: Cudinamarca. 2015.

[16] Observatorio Nacional de Seguridad Vial. Boletín estadístico Colombia enero - diciembre 2017–2018. Accessed Dec 2018.

